# Development and characterization of simple sequence repeat markers for the invasive tetraploid waterweed *Elodea nuttallii* (Hydrocharitaceae)

**DOI:** 10.1002/aps3.1146

**Published:** 2018-05-09

**Authors:** Isabell Weickardt, Andreas Zehnsdorf, Walter Durka

**Affiliations:** ^1^ Centre for Environmental Biotechnology Helmholtz Centre for Environmental Research–UFZ Permoserstraße 15 D‐04318 Leipzig Germany; ^2^ Department of Community Ecology Helmholtz Centre for Environmental Research–UFZ Theodor‐Lieser‐Straße 4 D‐06120 Halle Germany

**Keywords:** clonality, *Elodea canadensis*, *Elodea nuttallii*, Hydrocharitaceae, invasive species

## Abstract

**Premise of the Study:**

To enhance the understanding of the recent invasion process of the clonal waterweed *Elodea nuttallii* (Hydrocharitaceae), analyses of population structure and genotypic diversity need to be undertaken, for which genetic markers are needed.

**Methods and Results:**

High‐throughput sequencing of DNA enriched for microsatellites was used to develop 24 loci that were characterized in *E. nuttallii*, 21 of which were polymorphic, with the number of alleles ranging from two to 10. In two populations, expected heterozygosity ranged among loci between zero and 0.796. In the congener *E. canadensis*, all markers yielded PCR products, 19 of which were polymorphic, with two to nine alleles and expected heterozygosity ranging from zero to 0.690 in two populations.

**Conclusions:**

The markers described should be useful for future studies of population structure and clonal diversity of *E. nuttallii* as well as *E. canadensis* in their native and invasive range.

Western waterweed (*Elodea nuttallii* (Planch.) H. St. John, Hydrocharitaceae) is a tetraploid, dioecious, submerged freshwater macrophyte native to North America that is invasive in Europe and Japan. In its invasive range, almost exclusively female plants have been found and the reproduction is primarily vegetative (Cook and Urmi‐König, 1985). In Europe, it was first found in 1939 in Belgium and has quickly spread to 19 other European countries (Cook and Urmi‐König, 1985; Hussner, 2012). Because it can form dense dominant stands, *E. nuttallii* constrains water flow in rivers and the recreational use of lakes. Furthermore, the aggressive growth of this species may influence abiotic factors, outcompete native plants, change the makeup of the aquatic vegetation, and thus impact fish occurrence (Carey et al., 2016). Because no effective, species‐specific management options are known (Zehnsdorf et al., 2015), more information about the species’ biology and population structure is urgently needed. The analysis of genetic variation and population structure can give insights into reproduction mode, dispersal patterns, and the invasion process in general and may allow the identification of source regions in the native range (e.g., Durka et al., 2005; Voss et al., 2012).

Microsatellite markers are available for *E. canadensis* Michx. (Huotari et al., 2010); however, cross‐species amplification in *E. nuttallii* had not been tested (T. Huotari and H. Korpelainen, University of Helsinki, Helsinki, Finland, personal communication). Moreover, 60% of these markers only amplified in *E. canadensis* samples from invasive but not from native populations (Huotari et al., 2011), and preliminary tests revealed no or nonreproducible amplification products in our samples from both *E. canadensis* and *E. nuttallii*. Additionally, the available markers have limited power to discriminate clones because of low number of alleles (mean number of alleles = 2.8; Huotari et al., 2011) and their limited number (*N* = 10). Therefore, we developed new markers specifically for *E. nuttallii*. We demonstrate their utility for native and invasive samples as well as trans‐species amplification in *E. canadensis*, thus allowing for comparative analyses of clonal variation between native and invasive range and among species.

## METHODS AND RESULTS

Total genomic DNA from *E. nuttallii* collected in Großer Goitzschesee, Germany (51°37′12″N, 12°23′59″E), was isolated using the cetyltrimethylammonium bromide (CTAB) extraction procedure of Doyle (1991) and sent to GenoScreen (Lille, France). One microgram of DNA was used for the development of microsatellites through 454 GS‐FLX Titanium pyrosequencing (Roche Applied Science, Basel, Switzerland) of enriched DNA libraries following Malausa et al. (2011). Briefly, total DNA was mechanically fragmented and enriched for AG, AC, AAC, AAG, AGG, ACG, ACAT, and ATCT repeat motifs. Enriched fragments were subsequently amplified. PCR products were purified and quantified, and GsFLX libraries were then prepared following the manufacturer's protocols and sequenced on a GsFLX PTP (Roche Applied Science). Using QDD (Meglécz et al., 2010), adapters and vectors were removed, microsatellites were detected, their redundancy and association with mobile elements were tested, sequences with target microsatellites were selected, and primers were designed.

Among 2815 sequences comprising a microsatellite motif, 168 bioinformatically validated primer sets were designed on perfect motif microsatellites. We chose 41 primer pairs based on their motif, repeat number, and length of expected amplification product. We aimed to equally represent dinucleotide and trinucleotide repeats as well as short (90–140 bp), medium (140–190 bp), and long (190–250 bp) amplification products. Furthermore, microsatellites with high repeat numbers were favored because they often display a higher mutation rate.

These 41 primer pairs were ordered from Eurofins (Ebersberg, Germany) and were screened using the method of Schuelke (2000), i.e., adding CAG‐ or M13R‐tags to forward primers, adding GTTT‐tags to reverse primers (Brownstein et al., 1996), and using fluorescent‐labeled CAG and M13R primers. The PCR reaction contained 5 μL QIAGEN Multiplex PCR Master Mix (QIAGEN, Hilden, Germany), 1 μL of 2.5 μM tagged forward primer and 0.5 μM reverse primer, 1 μL of 2.5 μM fluorescent‐labeled CAG‐ or M13R‐tag, 2 μL of ultrapure water (Carl Roth GmbH, Karlsruhe, Germany), and 1 μL of DNA (~400 ng/μL). A touchdown protocol was used with cycles as follows: 95°C for 15 min; 20 cycles of 94°C for 30 s, 60°C for 30 s (decreasing 0.5°C per annealing cycle), and 72°C for 90 s; 20 cycles of 94°C for 30 s, 50°C for 30 s, and 72°C for 90 s; and a final elongation step of 10 min at 72°C. PCR products (1 μL) were multiplexed into four mixes (Table 1) with 10 μL of Hi‐Di Formamide (Thermo Fisher Scientific, Waltham, Massachusetts, USA) for fragment length analysis on an ABI 3130xl genetic analyzer (Thermo Fisher Scientific) with GeneScan 500 LIZ Size Standard (Thermo Fisher Scientific). Genotyping was performed using GeneMapper Software 5 (Thermo Fisher Scientific), allowing for a maximum of four alleles, assuming tetraploidy (Cook and Urmi‐König, 1985; Di Nino, 2008).

**Table 1:** Characteristics of 24 microsatellite markers developed in *Elodea nuttallii* and cross‐species amplification in *E. canadensis*

Locus	Primer sequences (5′–3′)^a^	Repeat motif	Fluorescent dye	PCR mix	Fragment analysis mix	*E. nuttallii* (*N* = 262)^b^				*E. canadensis* (*N* = 92)^b^				GenBank accession no.
						Allele size range (bp)	*A*	*A*_max_	*P*_NA_	Allele size range (bp)	*A*	*A*_max_	*P*_NA_	
Enu01	F: *CAGTCGGGCGTCATCA*GGTGGAAGATGAGCCGTAAG	(GA)_7_	NED	e	4	119	1	1	0	119	1	1	0	MG272338
	R: *GTTT*GTCGAGTAGGCACGTCGAA													
Enu04	F: *GGAAACAGCTATGACCAT*TAGGCTCTCATGCCTTTCC	(TC)_10_	PET	b	1	121–129	4	3	0	123–129	3	1	1.1	MG272339
	R: *GTTT*CGGTAGTCAGCAGTGGTGGT													
Enu05	F: *CAGTCGGGCGTCATCA*GATCTGGACCCAAAGCGAA	(TC)_7_	FAM	—	3	93–128	10	3	0	93–116	3	2	0	MG272340
	R: *GTTT*CAGATAGAGTGGTCTTCGCCA													
Enu06	F: *GGAAACAGCTATGACCAT*CCTTCTTGCTAGGGAAGAATATC	(TC)_8_	VIC	—	2	101–107	3	2	0	101–109	5	3	0	MG272341
	R: *GTTT*AGCCACTGCATCATGTCTTG													
Enu07	F: *CAGTCGGGCGTCATCAG*GTAGGTAGTCGAACACCAACATA	(CT)_8_	VIC	a	1	106–114	4	3	0	110–114	3	3	0	MG272342
	R: *GTTT*CATATGTACACCGAGATTGCAC													
Enu09	F: *GGAAACAGCTATGACCAT*CGAGTTGGCACAAGTAGGGT	(TCT)_9_	VIC	—	4	108–126	7	4	0	108–129	6	4	0	MG272343
	R: *GTTT*CCTCCAAAGAAACGCAAATC													
Enu10	F: *CAGTCGGGCGTCATCA*GAAGAGGTAGAACTCTACAATGAGGG	(GAG)_7_	NED	d	3	106–118	5	3	0.8	109–118	3	2	2.2	MG272344
	R: *GTTT*GGTCAGCACATGTCTCCTTTT													
Enu12	F: *CAGTCGGGCGTCATCA*GCTCTCCTCCCTTCTTGT	(TTC)_11_	NED	c	2	104–128	9	4	0	104–119	4	3	0	MG272345
	R: *GTTT*GGGAAGACCCATACCTTGCT													
Enu13	F: *GGAAACAGCTATGACCAT*CTTTCCACCCAAGCCCTAC	(CTT)_9_	VIC	—	3	108–135	9	4	0	111–135	7	3	0	MG272346
	R: *GTTT*GACAGTCCGATGGTGAAGGT													
Enu15	F: *CAGTCGGGCGTCATCA*GAGGTGTAGGGCTCTCAGTT	(TC)_6_	FAM	—	4	150–160	3	3	0	138–160	3	2	0	MG272347
	R: *GTTT*CTTTGGGGTCTAGGGAGGG													
Enu19	F: *CAGTCGGGCGTCATCA*TTACTAGCTTCGACACGCGA	(GA)_6_	NED	c	2	155–162	3	2	0.4	155–162	3	2	91.3	MG272348
	R: *GTTT*GGGAAGTGAGTTGAACGGAA													
Enu20	F: *GGAAACAGCTATGACCAT*AGCTTAGGGTTTGGTCCCAT	(CT)_6_	PET	d	3	156–164	5	3	0	156–158	2	1	89.1	MG272349
	R: *GTTT*AAGCATAAAAGCGAAAGCCA													
Enu21	F: *CAGTCGGGCGTCATCA*GCAACTGCTCGTTCTTCCAT	(TC)_6_	NED	b	1	132–170	7	3	1.5	150–170	4	3	4.4	MG272350
	R: *GTTT*GAGGCTCTGGACTCCAATCA													
Enu22	F: *CAGTCGGGCGTCATCA*GTCTCCTCCATTGATGCTCC	(TCC)_7_	FAM	f	1	141	1	1	0	141	1	1	5.4	MG272351
	R: *GTTT*CAAGAAAAGACCCCAAGGA													
Enu24	F: *CAGTCGGGCGTCATCA*CCACAGTTGGTCACTACTTCCA	(CTT)_9_	VIC	a	1	160	1	1	0	160	1	1	0	MG272352
	R: *GTTT*GAAGCTGCAGAAGAAACAAACA													
Enu26	F: *CAGTCGGGCGTCATCA*TATGTTTGGGCGATGTTTGA	(TTC)_11_	FAM	—	2	121–149	10	4	0	121–146	3	2	0	MG272353
	R: *GTTT*GTCGAGTGGGGTCTAAGG													
Enu28	F: *CAGTCGGGCGTCATCA*GTCACGATCCCCTTCTTCAA	(TCT)_6_	NED	e	4	149–158	3	3	3.8	149–158	3	2	4.4	MG272354
	R: *GTTT*CCTTATGCAAAGGAGGAATCA													
Enu30	F: *GGAAACAGCTATGACCAT*GATCGGAGATGAGGGAATGA	(GA)_8_	PET	c	2	218–228	3	2	3.4	222	1	1	97.8	MG272355
	R: *GTTT*CGCCATAGGCACGATGAT													
Enu35	F: *CAGTCGGGCGTCATCA*GAGTGAGGGATCGGGGATAG	(GGA)_6_	FAM	f	1	188–200	4	2	0.4	188–203	4	3	0	MG272356
	R: *GTTT*CTGCGACCATCCTACTGCTT													
Enu36	F: *GGAAACAGCTATGACCAT*AAACAACGAGAAGAAGCGGA	(GAA)_7_	PET	e	4	184–188	2	2	1.1	185	1	1	1.1	MG272357
	R: *GTTT*CGTAGCTCGTTCCATTTTCC													
Enu37	F: *CAGTCGGGCGTCATCA*CCATTTTCGTCCTTCTTCCA	(TTC)_11_	VIC	a	1	185–216	9	3	1.1	185–198	3	2	91.3	MG272358
	R: *GTTT*GACCTTCCTCCTTGGTG													
Enu38	F: *GGAAACAGCTATGACCAT*ACGGACGTTGGACCTCTATG	(AGT)_15_	PET	b	1	180–219	9	3	0	183–216	9	4	0	MG272359
	R: *GTTT*GTGATTGCTCCTTTTGTGG													
Enu39	F: *CAGTCGGGCGTCATCA*CGATCGTCAGAGACCTCACA	(CTT)_10_	NED	d	3	214–232	7	4	3.0	220–232	5	3	0	MG272360
	R: *GTTT*ATCACGTGAAATGCCAGTCA													
Enu41	F: *CAGTCGGGCGTCATCA*GCGAGCAACGAGATGAATTT	(CTT)_14_	NED	b	1	230–257	8	4	2.0	223–254	9	4	3.3	MG272361
	R: *GTTT*AATTACATTGGCGCGGTATC													

Note

— = singleplex reaction; *A* = number of alleles; *A*_max_ = maximal number of alleles detected per individual; *N* = number of individuals sampled; *P*_NA_ = percentage homozygous null alleles.

a Forward primer sequences include CAG and M13R tags and reverse primers include GTTT PIG‐tails (in italics).

b Geographic coordinates for the populations and voucher information are given in Appendix 1.

The 41 primers were tested for polymorphism on 40 individuals from eastern Germany, and 24 loci yielded PCR products in the expected size range (Table 1). Sequences containing these primers were deposited in the National Center for Biotechnology Information's GenBank database. The 24 markers were amplified in six multiplex and six singleplex reactions (Table 1). In case no PCR product was detected, at least two replicate analyses were performed; if no PCR product was found in the replicate analyses, the presence of a fixed null allele was assumed. Because of clonal reproduction and invasive spread, single populations harbored only a small amount of total allelic variation. Therefore, to assess genetic variation at the population level, we analyzed two well‐sampled invasive populations for both *E. nuttallii* and *E. canadensis*. For these, we report the number of genotypes (*N*_gt_) and used GenoDive version 2.0b23 (Meirmans and Van Tienderen, 2004) to calculate values of observed heterozygosity (*H*_o_) and expected heterozygosity (*H*_e_) correcting for unknown allele dosage of polyploids. To assess allelic variation at the species level, we included additional samples from other native and invasive sites, totaling 262 ramets from 53 sites in *E. nuttallii* and 92 ramets from 30 sites in *E. canadensis* (Appendix 1).

In *E. nuttallii*, 21 microsatellites were polymorphic, with the number of alleles ranging from two to 10 (average 5.3), totaling 127 alleles (Table 1). At population level, moderate levels of genetic diversity were found in two populations (mean *H*_e_ = 0.350 and 0.376), but genotypic diversity was either very low (mean *N*_gt_ = 1.04) or high (*N*_gt_ = 3.67; Table 2).

**Table 2:** Genotypic and genetic variation of 24 newly developed microsatellites at population and overall level in two invasive populations each of *Elodea nuttallii* and *E. canadensis*.^a^

Locus	*E. nuttallii*						*E. canadensis*					
	Cospudener See (*N* = 21)			Großer Goitzschesee (*N* = 43)			Gravel pit Kleinpösna (*N* = 27)			Parthe River (*N* = 11)		
	*N*_gt_	*H*_o_	*H*_e_	*N*_gt_	*H*_o_	*H*_e_	*N*_gt_	*H*_o_	*H*_e_	*N*_gt_	*H*_o_	*H*_e_
Enu01	1	0	0.000	1	0	0.000	1	0	0.000	1	0	0.000
Enu04	1	0	0.000	4	0.095	0.343	1	0	0.000	1	0	0.000
Enu05	1	0	0.000	3	0.048	0.084	1	0	0.000	1	0	0.000
Enu06	1	0	0.000	2	0.024	0.015	2	0	0.074	2	0	0.533
Enu07	1	1	0.677	1	1	0.506	1	1	0.675	1	1	0.690
Enu09	1	1	0.677	7	1	0.715	1	0	0.000	1	0	0.000
Enu10	1	1	0.512	2	1	0.506	1	0	0.000	1	0	0.000
Enu12	1	1	0.677	9	1	0.720	1	1	0.509	1	1	0.526
Enu13	1	1	0.512	3	1	0.570	4	1	0.614	2	0.8	0.490
Enu15	1	1	0.512	7	0.667	0.616	1	0	0.000	1	0	0.000
Enu19	1	0	0.000	3	0.357	0.385	—	—	—	—	—	—
Enu20	1	1	0.512	2	0.857	0.485	—	—	—	—	—	—
Enu21	1	1	0.512	2	0.976	0.505	1	0	0.000	1	0	0.000
Enu22	1	0	0.000	1	0	0.000	1	0	0.000	1	0	0.000
Enu24	1	0	0.000	1	0	0.000	1	0	0.000	1	0	0.000
Enu26	1	1	0.759	12	1	0.796	1	0	0.000	1	0	0.000
Enu28	1	0	0.000	1	0	0.000	1	1	0.509	1	1	0.526
Enu30	1	0	0.000	2	0.095	0.060	—	—	—	—	—	—
Enu35	1	0	0.000	1	0	0.000	1	0	0.000	2	0.8	0.490
Enu36	1	1	0.512	2	0.619	0.378	1	0	0.000	1	0	0.000
Enu37	1	1	0.512	5	0.81	0.479	—	—	—	—	—	—
Enu38	2	1	0.677	4	0.952	0.604	1	1	0.675	2	1	0.689
Enu39	1	1	0.677	5	0.929	0.563	2	0.185	0.127	3	1	0.608
Enu41	1	1	0.677	8	0.929	0.688	1	1	0.675	1	1	0.690
Average	1.04	0.583	0.350	3.67	0.557	0.376	1.25	0.309	0.193	1.30	0.38	0.262

Note

— = no amplification; *H*_e_ = expected heterozygosity corrected for allele dosage; *H*_o_ = observed heterozygosity; *N* = number of individuals sampled; *N*_gt_ = number of genotypes.

a Geographic coordinates for the populations and voucher information are given in Appendix 1.

Cross‐species amplification in *E. canadensis* was successful, revealing 19 polymorphic loci with two to nine alleles per locus (average 3.6) and 87 alleles in total (Table 1). However, four loci had high null allele frequencies. At population level, low levels of both genetic diversity (mean *H*_e_ = 0.193 and 0.262; Table 2) and genotypic diversity (mean *N*_gt_ = 1.25 and 1.30) were found in two populations. In both species, the number of alleles detected per individual and locus ranged between one and four, as expected for a tetraploid species (Table 1).

## CONCLUSIONS

The microsatellite markers developed for *E. nuttallii* were also proved to be useful in *E. canadensis*. Allelic diversity was generally higher in invasive populations of *E. nuttallii* compared to invasive populations of *E. canadensis*. This suggests that different processes may drive the invasion of these two morphologically and ecologically highly similar species. The new markers will be useful for further analysis of clonal diversity and genetic structure of *E. nuttallii* and *E. canadensis* and, in particular, to investigate their European invasion history.

## APPENDIX 1 Locality and voucher information for *Elodea* populations used in this study.

**Table:**

Species	Country, municipality/state, waterbody	Geographic coordinates	No. of ramets	Voucher^a^
*E. canadensis* Michx.	Germany, Bavaria, Chiemsee	47°52′21″N, 12°27′36″E	1	EMT_41
*E. canadensis*	Germany, Bavaria, Ferchensee	47°26′19″N, 11°12′49″E	1	EMT_42
*E. canadensis*	Germany, Bavaria, Froschhauser See	47°41′13″N, 11°13′28″E	1	EMT_37
*E. canadensis*	Germany, Bavaria, Moosach	48°23′38″N, 11°43′27″E	1	EMT_38
*E. canadensis*	Germany, Bavaria, Starnberge See	47°54′45″N, 11°18′25″E	1	EMT_43
*E. canadensis*	Germany, Lower Saxony, Hauptkanal	52°53′20″N, 8°58′53″E	1	EMT_28
*E. canadensis*	Germany, North Rhine‐Westphalia, Diersfordter Waldsee	51°41′46″N, 6°31′54″E	1	EMT_70
*E. canadensis*	Germany, North Rhine‐Westphalia, Tenderingsee	51°35′43″N, 6°43′30″E	1	EMT_67
*E. canadensis*	Germany, Saxony, Delinkateich	51°24′25″N, 14°45′00″E	1	EMT_11
*E. canadensis*	Germany, Saxony, gravel pit Kleinpösna	51°18′43″N, 12°31′51″E	27	EMT_130
*E. canadensis*	Germany, Saxony, Kleinliebenau	51°22′01″N, 12°13′31″E	1	EMT_328
*E. canadensis*	Germany, Saxony, Kulkwitzer See	51°17′52″N, 12°15′14″E	1	EMT_51
*E. canadensis*	Germany, Saxony, Cospudener See	51°17′06″N, 12°21′38″E	1	EMT_323
*E. canadensis*	Germany, Saxony, Leipzig	51°19′41″N, 12°17′27″E	2	EMT_324
*E. canadensis*	Germany, Saxony, Nangteich	51°24′30″N, 14°45′01″E	1	EMT_329
*E. canadensis*	Germany, Saxony, Parthe	51°22′09″N, 12°24′47″E	11	EMT_266
*E. canadensis*	Germany, Saxony‐Anhalt, Hasselvorsperre	51°42′33″N, 10°49′51″E	1	EMT_50
*E. canadensis*	Germany, Saxony‐Anhalt, Mulde	51°40′51″N, 12°17′41″E	2	EMT_249
*E. canadensis*	Germany, Saxony‐Anhalt, Raßnitzer See	51°22′0″N, 12°4′41″E	5	EMT_87
*E. canadensis*	Norway, Buskerud, Tyrifjorden	59°57′51″N, 9°59′47″E	1	EMT_317
*E. canadensis*	Austria, Salzkammergut, Grundlsee	47°37′58″N, 13°51′52″E	1	EMT_39
*E. canadensis*	Peru, Cusco, Oropesa	−13°33′37″N, −71°51′40″E	1	EMT_338
*E. canadensis*	Purchased online (Nymphaion)		6	EMT_347
*E. canadensis*	Slovenia, Podravska, Ptujsko jezero	46°23′14″N, 15°54′25″E	1	EMT_307
*E. canadensis*	USA, Alaska, Alexander Lake	61°44′38″N, −150°53′31″E	1	EMT_61
*E. canadensis*	USA, Maryland, Conococheague Creek	39°41′38″N, −77°48′34″E	2	EMT_308
*E. canadensis*	USA, New York, Collins Lake	42°49′38″N, −73°57′15″E	6	EMT_284
*E. canadensis*	USA, New York, Lake Ontario	43°18′43″N, −77°43′13″E	9	EMT_285
*E. canadensis*	USA, New York, Niagara River	43°06′26″N, −78°58′41″E	1	EMT_300
*E. canadensis*	USA, Pennsylvania, Erie See	42°07′41″N, −80°08′33″E	1	EMT_303
*E. nuttallii* (Planch.) H. St. John	Germany, Baden‐Württemberg, Bodensee	47°44′59″N, 9°07′54″E	3	EMT_359
*E. nuttallii*	Germany, Baden‐Württemberg, Brigach	47°59′51″N, 8°27′48″E	1	EMT_32
*E. nuttallii*	Germany, Baden‐Württemberg, Donau	47°54′57″N, 8°34′9″E	2	EMT_30
*E. nuttallii*	Germany, Baden‐Württemberg, Kapuzinergraben	48°17′26″N, 7°47′46″E	3	EMT_354
*E. nuttallii*	Germany, Baden‐Württemberg, Rhein	48°12′26″N, 7°39′31″E	14	EMT_356
*E. nuttallii*	Germany, Bavaria, Chiemsee	47°51′49″N, 12°24′58″E	1	EMT_40
*E. nuttallii*	Germany, Bavaria, Ilm	48°25′58″N, 11°23′55″E	1	EMT_35
*E. nuttallii*	Germany, Bavaria, Kleine Vils	48°29′8″N, 12°18′18″E	1	EMT_36
*E. nuttallii*	Germany, Bavaria, Starnberge See	47°54′53″N, 11°17′41″E	3	EMT_34
*E. nuttallii*	Germany, Berlin, Tegeler See	52°35′33″N, 13°15′44″E	1	EMT_274
*E. nuttallii*	Germany, North Rhine‐Westphalia, Baldeneysee	51°23′56″N, 7°02′56″E	1	EMT_208
*E. nuttallii*	Germany, North Rhine‐Westphalia, Hürther Waldsee	50°52′26″N, 6°50′36″E	1	EMT_64
*E. nuttallii*	Germany, North Rhine‐Westphalia, Kemnader See	51°25′20″N, 7°16′00″E	4	EMT_213
*E. nuttallii*	Germany, North Rhine‐Westphalia, Lippe	51°41′43″N, 7°50′28″E	1	EMT_254
*E. nuttallii*	Germany, North Rhine‐Westphalia, Rotbach	51°34′24″N, 6°47′40″E	1	EMT_237
*E. nuttallii*	Germany, Rhineland‐Palatinate, Dreifelder Weiher	50°35′27″N, 7°49′33″E	1	EMT_66
*E. nuttallii*	Germany, Rhineland‐Palatinate, Laacher See	50°24′48″N, 7°16′20″E	1	EMT_69
*E. nuttallii*	Germany, Rhineland‐Palatinate, Rhein	49°17′9″N, 8°27′21″E	2	EMT_65
*E. nuttallii*	Germany, Rhineland‐Palatinate, Schäferweiher	50°33′40″N, 7°44′01″E	1	EMT_68
*E. nuttallii*	Germany, Saxony, Berzdorfer See	51°06′13″N, 14°58′35″E	1	EMT_45
*E. nuttallii*	Germany, Saxony, Chausseeteich	51°24′38″N, 14°47′19″E	2	EMT_268
*E. nuttallii*	Germany, Saxony, Cospudener See	51°15′37″N, 12°20′19″E	21	EMT_252
*E. nuttallii*	Germany, Saxony, gravel pit Kleinliebenau	51°22′08″N, 12°11′59″E	3	EMT_337
*E. nuttallii*	Germany, Saxony, gravel pit Kleinpösna	51°18′35″N, 12°31′45″E	23	EMT_104
*E. nuttallii*	Germany, Saxony, Heideteich	51°24′36″N, 14°45′33″E	2	EMT_23
*E. nuttallii*	Germany, Saxony, Leipzig	51°15′45″N, 12°21′21″E	2	EMT_321
*E. nuttallii*	Germany, Saxony, Mulde	51°31′40″N, 12°36′28″E	1	EMT_275
*E. nuttallii*	Germany, Saxony, Mylau	50°37′12″N, 12°16′33″E	1	EMT_331
*E. nuttallii*	Germany, Saxony, Parthe	51°21′40″N, 12°24′28″E	9	EMT_266
*E. nuttallii*	Germany, Saxony, Unterer Oberteich	51°24′36″N, 14°47′17″E	3	EMT_21
*E. nuttallii*	Germany, Saxony, Weiße Elster	51°12′19″N, 12°18′16″E	20	EMT_234
*E. nuttallii*	Germany, Saxony, Weiße Schöps	51°22′57″N, 14°44′01″E	1	EMT_335
*E. nuttallii*	Germany, Saxony, Winterteich	51°23′16″N, 14°51′42″E	1	EMT_267
*E. nuttallii*	Germany, Saxony, Zwenkauer See	51°12′57″N, 12°21′15″E	12	EMT_261
*E. nuttallii*	Germany, Saxony‐Anhalt, Elbe	52°02′01″N, 1°52′39″E	4	EMT_256
*E. nuttallii*	Germany, Saxony‐Anhalt, Großer Goitzschesee	51°37′12″N, 12°23′59″E	43	EMT_185
*E. nuttallii*	Germany, Saxony‐Anhalt, Mulde	51°35′44″N, 12°30′03″E	7	EMT_204
*E. nuttallii*	Germany, Saxony‐Anhalt, Muldestausee	51°37′16″N, 12°25′01″E	15	EMT_201
*E. nuttallii*	Germany, Saxony‐Anhalt, Raßnitzer See	51°21′59″N, 12°4′59″E	19	EMT_76
*E. nuttallii*	Germany, Saxony‐Anhalt, Saale	51°34′52″N, 11°48′46″E	1	EMT_242
*E. nuttallii*	Germany, Saxony‐Anhalt, Seelhausener See	51°35′19″N, 12°26′27″E	9	EMT_56
*E. nuttallii*	Peru, Cusco, Oropesa	−13°33′37″N, −71°51′40″E	1	EMT_340
*E. nuttallii*	Purchased online (Nymphaion)		4	EMT_343
*E. nuttallii*	Slovenia, Podravska, Drava	46°24′17″N, 16°08′59″E	1	EMT_306
*E. nuttallii*	Slovenia, Oresje	45°53′11″N, 15°16′07″E	1	EMT_305
*E. nuttallii*	USA, Alaska, Potter Marsh	61°03′47″N, −149°47′55″E	1	EMT_60
*E. nuttallii*	USA, Maryland, Potomac River	39°36′27″N, −77°58′08″E	1	EMT_310
*E. nuttallii*	USA, New York, Collins Lake	42°49′38″N, −73°57′15″E	1	EMT_284.2
*E. nuttallii*	USA, New York, Lake Ontario	43°21′22″N, −77°56′16″E	1	EMT_298
*E. nuttallii*	USA, New York, Niagara River	43°03′36″N, −78°58′41″E	2	EMT_301
*E. nuttallii*	USA, Pennsylvania, Lake Erie	42°07′41″N, −80°08′25″E	1	EMT_302
*E. nuttallii*	USA, Pennsylvania, Quaker Lake	41°58′39″N, −75°55′08″E	2	EMT_314
*E. nuttallii*	USA, Virginia, Gunston Cove	38°40′30″N, −77°09′20″E	3	EMT_311

a Vouchers are deposited at the Helmholtz Centre for Environmental Research, Permoserstraße 15, 04318 Leipzig, Germany.
